# Understanding the lived experience of idiopathic pulmonary fibrosis and how this shapes views on home-based pulmonary rehabilitation in Delhi, India

**DOI:** 10.1177/14799731241258216

**Published:** 2024-05-24

**Authors:** Humaira Hanif, Obaidullah Ahmed, James Manifield, Rubia Ishrat, Ilaria Pina, Zahira Ahmed, Mohd Shibli, Dominic Malcolm, Deepak Talwar, Sally J. Singh, Mark W Orme

**Affiliations:** 1 Metro Centre for Respiratory Disease, 80199 Metro Hospital and Heart Institute, Noida, India; 2 Centre for Exercise and Rehabilitation Science, Leicester Biomedical Research Centre (BRC) – Respiratory, University Hospitals of Leicester NHS Trust, National Institute for Health and Care Research (NIHR), Leicester, UK; 3 Department of Respiratory Sciences, 4488 University of Leicester, Leicester, UK; 4 School of Sport, Exercise and Health Sciences, 5156 Loughborough University, Loughborough, UK

**Keywords:** Home-based pulmonary rehabilitation, idiopathic pulmonary fibrosis, interstitial lung disease, self-management, chronic respiratory diseases, qualitative research

## Abstract

**Objectives:**

Pulmonary Rehabilitation (PR) is a high-impact intervention for individuals with idiopathic pulmonary fibrosis (IPF) but access is limited in India. PR barriers include distance to travel, lack of service provision and lack of healthcare professionals to deliver PR, thus it is disproportionate to the immense burden of IPF in India. We explored the lived experiences of people living with IPF, family caregivers (CGs) and healthcare workers (HCWs) as well as their views towards home-based PR (HBPR) in Delhi, India.

**Methods:**

A qualitative study using semi-structured interviews with individuals with IPF (n = 20), CGs (n = 10) and HCWs (n = 10) was conducted. Data were analysed using codebook thematic analysis.

**Results:**

Three major themes were generated: (i) Health impact, which included pathophysiological changes, range of symptoms experienced, disease consequences and impact of comorbidities; (ii) Disease management, which described strategies to control the progression and overall management of IPF, such as medications and exercises; (iii) Mode of Pulmonary Rehabilitation, which described perceptions regarding HBPR, comparisons with centre-based programmes, and how HBPR may fit as part of a menu of PR delivery options.

**Conclusion:**

People living with IPF, family caregivers and healthcare workers were positive about the potential implementation of HBPR and suggested the development of a paper-based manual to facilitate HBPR over digital/online approaches. The content of HBPR should be sensitive to the additional impact of non-IPF health issues and challenges of reduced interactions with healthcare professionals.

## Introduction

Idiopathic pulmonary fibrosis (IPF) is a chronic, progressive interstitial pneumonia that often presents as a chronic cough and dyspnoea upon exertion.^1^ Global prevalence and incidence have been estimated in the range of 0.33–4.51 and 0.09–1.30 per 10,000 persons, respectively.^2^ IPF is a common entity among diffuse parenchymal lung disease cases in India,^3^ with the estimated national prevalence being 5.8-11.6 per 100,000.^4^ IPF is detrimental to health-related quality of life^5^ and economically burdensome for individuals and society.^6^ Symptoms of IPF can lead to hypoxia and dyspnoea due to ventilatory dysfunction which is a key contributing factor of depression and poor functional capacity.^7^

Pulmonary rehabilitation (PR) is recommended for people living with IPF to enhance their functional capacity and improve quality of life.^8^ Despite evidence of its benefits for IPF^9^ and its recommendation in guidelines,^10^ the availability of PR in low- and middle-income countries (LMIC), including India, remains limited.^11^

Given that India is a populous country, there is a clear need for different modes of PR delivery to offer a menu of choice to people with IPF and other lung conditions. Appropriately tailoring PR to individuals and the local context may help optimise uptake and completion. Home-based PR (HBPR) may be a particularly suitable mode of PR in India, including for those individuals who would otherwise find it challenging to attend a centre-based programme, such as those with significant household responsibilities, in employment, without access to transport, residing in remote areas and/or without adequate support from family.^10^

The lived experiences of people with IPF in India has not been well explored and their views on PR are largely unknown. Therefore, the aims were to (i) explore the lived experiences of individuals with IPF along with the experiences of family caregivers and health care workers in Delhi, India and (ii) explore consideration for PR in a home-based setting within this context.

## Methods

A qualitative study using semi-structured interviews was conducted. The study was approved by the ethics review committees of Metro Hospitals & Heart Institute, Noida, India (ref: 62/MERB/2021) and University of Leicester, UK (ref: 31989), as part of the National Institute for Health and Care Research Global RECHARGE project.^12^ This study is reported in accordance with the COnsolidated criteria for REporting Qualitative research (COREQ) guidelines^13^ (supplementary material 1).

### Participants

Three groups of participants were recruited for this study: people living with IPF, their caregivers (CGs) and healthcare workers (HCWs) involved in IPF patient care. A purposive sample was recruited to obtain the participation of a range of HCWs job roles, and people living with IPF of varying ages, genders, and previous PR experience. Further details are provided in supplementary material 2.

#### Inclusion criteria

Adults (aged ≥18 years) diagnosed with IPF as per ATS/ERS guidelines^14^ who were not required to have previous experience of PR. HCWs who had more than 1 year experience working directly with IPF patients (e.g., pulmonologists, physiotherapists, PR specialists, respiratory therapists, and respiratory care technicians). CG had to be taking care of a family member diagnosed with IPF to be eligible and it was not necessary that CG participation was contingent on the participation of their respective relative with IPF or vice versa. All participants had to be able to provide informed consent.

### Interview process

Semi-structured interviews were undertaken with all three participant groups, and were conducted from 1^st^ November 2021-17^h^ February 2022.

Topic guides for the interviews (supplementary material 3) were semi-structured, followed an iterative approach, and included prompts for certain questions (further details provided in supplementary material 2).

Participants were aware that the purpose of research was to explore the lived experiences and views towards HBPR. Questions varied between participant groups but common questions covered disease knowledge and management, need for PR, PR importance, delivery of PR, knowledge about HBPR, perceptions about HBPR including its advantages, challenges, and suggestions for key components and considerations of HBPR.

For participants that were unfamiliar with HBPR, a definition (adapted from previously published definition^10^) was provided prior to related questions: “*Home-based pulmonary rehabilitation is an intervention performed at home which includes exercise training, education, and behaviour change to improve the condition of people with chronic respiratory disease”.*

Interviews were digitally recorded and transcribed verbatim by the researchers. Descriptive and reflective field notes were taken by the interviewer after each interview.

### Data analysis

Codebook thematic analysis was conducted following the six phases of Braun and Clarke.^15^ Further details are provided in supplementary material 2.

A grounded theory approach was adopted, and inductive reasoning was used to generate theories from the data. This allowed us to compare the perspectives of the 3 participant groups, and due to the lack of prior relevant research exploring home-based PR in India, a deductive approach was deemed inappropriate.

## Results

A total of 40 interviews were conducted in person (IPF, *n* = 11; CG, *n* = 6; HCW, *n* = 9) or via telephone (IPF, *n* = 9; CG, *n* = 4; HCW, *n* = 1) due to distance to hospital or pandemic restrictions (Table 1). The average duration of interviews was 33 min (range: 10–55 min). Written informed consent was obtained from all participants.

**Table 1.** Characteristics of study participants.

Participant ID	Age (years)	Sex	Language interview conducted in	Previous PR participation
Individuals living with idiopathic pulmonary fibrosis (*n* = 20)				
P1001	52	Male	English	No
P1002	66	Male	English	Yes
P1003	65	Male	Hindi	No
P1004	51	Male	Mixed	No
P1005	70	Male	English	Yes
P1006	48	Female	Hindi	Yes
P1007	75	Male	Hindi	Yes
P1008	78	Male	Hindi	No
P1009	71	Male	Mixed	Yes
P1010	73	Male	English	No
P1011	55	Female	English	No
P1012	58	Female	Mixed	Yes
P1013	71	Male	English	No
P1014	74	Male	English	No
P1015	69	Male	Hindi	No
P1016	67	Male	English	Yes
P1017	81	Male	Hindi	No
P1018	77	Male	Hindi	No
P1019	64	Male	English	Yes
P1020	75	Male	Hindi	No

Participant ID	Relation to patient	Language interview conducted in
Caregivers (*n* = 10)		
C1001	Daughter-in-aw	English
C1002	Son	English
C1003	Daughter	Mixed
C1004	Wife	Hindi
C1005	Son	Mixed
C1006	Daughter	Mixed
C1007	Son	Mixed
C1008	Son	Hindi
C1009	Wife	Hindi
C1010	Son	English

Participant ID	Job title	Language interview conducted in
Healthcare workers (*n* = 10)		
H1001	Pulmonologist	English
H1002	Physiotherapist	Mixed
H1003	Pulmonary rehabilitation assistant	Hindi
H1004	Senior pulmonologist	English
H1005	Physiotherapist	English
H1006	Respiratory care technician	Hindi
H1007	Respiratory therapist	Mixed
H1008	Respiratory care technician	Hindi
H1009	Physiotherapist	English
H1010	Physiotherapist	Hindi

Three themes were generated from interview data: (i) Health impact (which focused on the impact of IPF symptoms on daily life, and the impact of comorbidities); (ii) Disease management (which included strategies to manage IPF, including medications, exercise participation and barriers to exercise) and (iii) Mode of PR (which determined perceptions regarding HBPR, including comparisons to centre-based programmes) (Table 2). Further quotes and interpretations are provided in supplementary material 4.

**Table 2.** Overview of themes and Sub-themes. Participant groups informing the development of each theme are identified in brackets.

Theme	Sub-themes
Health impact	a) IPF symptoms impact on daily life (Pt., CG, HCW)
	b) Comorbidities (Pt., CG, HCW)
Disease management	a) General IPF management (Pt., CG, HCW)
	b) PR and exercise as management strategies (Pt., CG, HCW)
	c) Barriers to exercise (Pt., CG)
Mode of PR	a) HBPR advantages (Pt., CG, HCW)
	b) Centre-based PR advantages (CG, HCW)
	c) HBPR challenges (Pt, HCW)
	d) Goals of service users (Pt.)
	e) HBPR suggestions (Pt., CG, HCW)

Pt., individuals with IPF; CG, caregiver; HCW, healthcare worker; PR, pulmonary rehabilitation; HBPR, home-based pulmonary rehabilitation.

### Health impact

#### Impact of IPF symptoms on daily life

Individuals with IPF reported experiencing symptoms such as coughing, oxygen desaturation, breathlessness, and their subsequent effects on daily activities, “*I started feeling breathless during toileting and bathroom activities. This problem has increased with time, and now I become breathless while standing, sitting als*o*” (P1018)*. The presence of these IPF symptoms were also acknowledged by HCW, highlighting that they may become more pronounced during daily activities, “*They feel trouble in oxygen saturation very often and dyspnoea as well which exaggerated with physical activities”* (H1002).

#### Comorbidities

Existing comorbidities were considered as contributing factors for symptom exacerbation. When these other conditions get worse, or an event occurs, then IPF symptoms also get worse, *“hepatitis B occurs due to which my few lungs’ medicines had stopped as it was harmful for liver which further leads to increase my breathlessness” (P1006).* For clinicians, IPF was seen as a causative factor for other comorbidities about which they discussed, *“these patients have lots of comorbidities like GERD [gastroesophageal reflux disease], pulmonary hypertension and depression associated with IPF” (H1004).*

### Disease management

#### General IPF management

Managing disease became crucial to relieve IPF symptoms and control disease progression. Participants talked about pharmaceutical treatments and their benefits on health, *“There was breathing problem and cough which […] were relieved by nebulisation and inhaler” (P1008).* HCWs also highlighted the importance of medication, along with rehabilitation, as vital IPF management strategies, *“Antifibrotic and rehabilitation both plays equal roles, 50% each, for the recovery of patients” (H1001).*

#### Pulmonary rehabilitation and exercise as management strategies

Individuals with IPF discussed current strategies relating to exercise that they employ to manage their symptoms. In some participants this involved avoiding physical activity altogether, *“now I do not exert myself very much. I am trying to do my various works in sitting position as I am unable to stand longer” (P1011).* Some HCWs explained how physical inactivity can further increase breathlessness and deconditioning leading to a vicious cycle of further inactivity and worsening symptoms. PR can reverse this cycle by increasing physical activity, reducing breathlessness and deconditioning, “*PR also helps in reducing the disease symptoms and frequent hospital admission of the patients […] it also relieves the medicines and its expense burden” (H1007).*

The benefits of exercise and PR were suggested by most CGs, “I feel exercise is the best way to relieve him from this situation. Medicines are really a part of his treatment but I think exercising daily in proper way will 80% help him a lot. (C1006). HCWs had previously observed improvements in IPF patient outcomes following PR/exercise, and were very much in favour of PR as an adjunct treatment, “We have the good PR centre itself which has been run by good doctors and trained physiotherapist. We have seen great improvements in patients and their survival time has also increase[d]” (H1001). The benefits of PR were echoed by individuals with IPF who had previous experience in these programmes, “After my diagnosis I was referred to PR centre, where my physiotherapist explained me about rehabilitation program which actually benefitted me” (P1019).

#### Barriers to exercise

Many individuals with IPF were unable to engage with centre-based PR (CBPR) sessions due to barriers such as dependency on family members, bad weather and other household duties. *“it’s difficult to go centre as I can’t drive now and my dependency is on my son who is occupied in work” (P1017).* HCWs suggested that a lack of patient knowledge surrounding PR could result in detrimental effects such as worsening symptoms and disease progression, “*most of the patients dependent on medicine for their treatment, they do not even know much about PR program. Lack of activity and exercise affect these patients on their physical and mental health and hence it progresses the disease” (H1007*).

### Home based PR/mode of PR

#### HBPR advantages

HBPR was considered by participants as a potential solution to some of the barriers to CBPR, “It is a very good option for those who are unable to take tele-rehabilitation or centre-based session… and in the coming time, due to pandemic or pollution, the lung patients may increase so it can benefit a large group” (P1006). Most CGs were very positive about HBPR; they discussed its advantages, with one patient’s son stating that HBPR may be a good initiative to make the individuals with IPF more independent. “Now on long run… we always want to make papa [Father] independent and for that home-based rehabilitation program will be booster” (C1002).

HCWs emphasised the importance of HBPR being a convenient option for many patients who are unable to participate in PR due to reasons such as musculoskeletal issues or travel distance, “*They also must do home-based rehabilitation if unable to visit centre. Some are unable to commute due to other orthopaedics condition so for them also it can work” (H1002);* “*and “If patient is not quite fit to come in the centre, then doing exercise with manual or recorded exercise videos is the best option” (H1005).*

#### Centre-based pulmonary rehabilitation (CBPR) advantages

There were varying opinions among HCWs regarding the most favourable mode of PR delivery. For some, CBPR was seen as more impactful due to the presence of HCWs and medical facilities, “*Because we have all those equipment at our centre, we also have doctors and technicians facilities so if any uncertainty happened, we can easily cope up with it and the patient will be assisted by whole team in our centre-based PR” (H1005).* Similarly, according to some CGs, CBPR was the preferred option, “*I would say centre-based rehabilitation there are experienced physiotherapist. They know best about the rehabilitation programme, because they [have] expertise in that field” (C1001).*

#### Challenges with HBPR

According to individuals with IPF, there could be various challenges relating to the unsupervised nature of HBPR, “*I personally feel there is lack of interaction with healthcare [workers] so if I do anything wrong or if I fall ill, there will be no medical facilities and doctor for my treatment” (P1002).* CGs reported a fear of acute exacerbation upon exertion, *“If tried to start exercise by chance then his oxygen level used to fall and the cough used to start and in a fear of increasing symptoms, he often quit to do exercise” (C1003).*

#### Goals of service users

Some individuals with IPF shared their desire to return to normal activities and improve fitness levels, *“patients want their normal life back. I want to do my daily activities smoothly without any trouble” (P1006).* Most individuals with IPF had a main goal to improve walking capacity, “*I expect breathlessness will be reduced […] Suppose today if I can walk for 100 feet then I should achieve 150 or 200 feet. I want my lungs to help more in walking”. (P1010).*

#### HBPR suggestion

Recommendations for the best implementation of HBPR were similar between individuals with IPF and CGs, and centred around more in-depth explanations of correct exercise techniques via a paper manual, online consultations, or video demonstration, *“Patients will do exercise while just reading HBPR manual which can affect exercise accuracy but with video call consultation for once in a while problem will be sorted out” (P1006)* and *“You can provide CD with videos of exercise. You can train the patient first and then give the exercise instructions booklet or you can also teach and train patients via video call” (C1007*).

CGs were keen to learn about the exercises, suggesting that they should be trained with specific PR exercises to help their family member during HBPR, “*Exercise booklet will become very helpful for us. I will read and train him by taking help from booklet” (C1005).* The importance of CG training in order to optimise intervention adherence and compliance was also highlighted by HCWs, *“But if the family members are very well trained, they are knowing and comfortable then we should go for home-based rehabilitation (H1001)* and *“if we guide them at the time of giving the HBPR manual then it will be easy for them to understand then they will read the techniques of exercises and medication details*.*” (H1003)*.

CGs also recommended the addition of educational components within HBPR to improve knowledge of disease management, “as this problem is new for me… I want you to add information about the possible consequences, progression and other things which will happen to my father in future so that I will become mentally prepare[d] and become aware of these changes” (C1010). Likewise, individuals with IPF believed that education and awareness programs need to be established to promote HBPR, “I think you should organise a seminar at least once in a year on pulmonary rehabilitation to connect your patients and doctors, so that they can share their experiences and interact with each other. I think by doing this patient will also learn newer methods of treatment and newer exercises” (P1019).

## Discussion

### Summary of main findings

This study is the first to explore the perspectives of individuals with IPF, CG and HCWs regarding HBPR for individuals living with IPF in India. The study identified the impacts of IPF on both individuals with IPF and family caregivers. Individuals with IPF reported a reduced quality of life due to the disease, and stated that comorbid medical conditions had a negative impact on their condition which was sometimes a barrier to exercise. Family caregivers reported the need to balance their professional life and patient care and/or hospital visits, contributing to their general support for the option of HBPR. There was a clear interest in, and demand for, HBPR among all three participant groups. Individuals with IPF expressed their strong desire for the development of HBPR to overcome the limitations of other PR delivery modes, such as travel and cost to attend CBPR programmes.

### Interpretation of findings

The positive views towards HBPR, specifically the flexible nature of the programme and reduced travel burden, were similar with those observed within a previous qualitative study conducted in Australia with people living with chronic obstructive pulmonary disease (COPD).^16^ Individuals with IPF in the present study felt that their independence would increase through HBPR compared to other modes of PR since they would not need to rely on their CG for hospital visits, or require help with smartphone technology for tele-rehabilitation. This was echoed by CGs, whose lives were also impacted by the time required to support their family member with managing their IPF. Consequently, with anticipated improvements in performing daily activities, participants were hopeful of gaining a greater sense of independence in their lives.

HCWs highlighted additional challenges in symptom management and improving functional capacity during HBPR compared with CBPR due to potentially reduced contact time. A previous comparison between these two modes of PR observed similar reductions in dyspnoea and enhanced functional capacity in both groups, although the study was inconclusive as to whether HBPR was superior or inferior to CBPR.^17^ HCWs acknowledged that HBPR would be a valuable alternative as part of a menu of PR options to improve access and uptake for people unable to attend CBPR. In order to overcome barriers such as travel, disability, geographical restrictions, and financial burden, it is vital to have innovative models in HBPR delivery.^10^

Suggestions for HBPR reported within this study included the use of a variety of mediums in which to provide guidance and information such as exercise videos, CDs or using a paper-based manual. The drawback of the digital option was that it was perceived as unsuitable for those who were non-/familiar with digital technologies, for whom a paper-based manual was seen as a convenient option. Participants suggested a hybrid approach, whereby a paper-based manual or exercise videos could be used alongside initial or weekly face-to-face sessions. This hybrid approach to PR involving a paper-based manual has previously been found to improve exercise performance, disease awareness and anxiety among people with COPD in the United Kingdom (UK).^18^ Having a HBPR option including a manual-based resource with clear instructions and visuals may facilitate adherence to correct exercise techniques and thus optimise uptake and completion of PR in the IPF population within India.

According to CG and HCWs, the development of a HBPR manual may enhance independence, overcome CG time management issues, and improve the knowledge of CGs enabling them to provide better support during exercises. HCWs discussed various advantages of this mode of PR, including the ability to monitor exercise progression as well as providing information related to disease, treatment, exercise, and exacerbation management. The responses from the present IPF participants were similar to experiences of people with COPD participating in self-management programmes in the UK, which found improvements in exercise performance, physical activity, and medication use.^19^ When interviewers asked about the best way to expand the delivery of PR in India, responses emphasised the need for improving education and awareness. Limited patient knowledge about PR has been found to be a significant barrier to access, uptake, and completion.^20^ Various resources, specifically relating to HBPR, were suggested to provide visual instructions (either in pictorial form or video representation) with minimal text and simple language.

It was important to participants that potential referrers to PR understand its value and appropriately inform individuals with IPF and their family members, allowing them to make an informed decision about participation. Participants also suggested having monthly interactions with HCWs to monitor their exercise techniques and answer any PR-related queries. These suggestions may help overcome some of the barriers to PR in LMIC highlighted both in the present study and in previous research.^21^

Both CGs and HCWs emphasised the need to be trained in the correct/safe exercise technique, allowing them to appropriately support exercises at home. CGs specifically suggested an initial tele-session or face-to-face consultation with HCW. This was echoed by HCWs who further suggested that CGs should have access to HCW contact number and necessary medical equipment (e.g., vital monitoring equipment and oxygen concentrator) when supporting HBPR. HCWs stated that the choice of PR mode should vary depending on individual patient scenarios, and that HBPR may not be an appropriate choice for individuals with locomotor difficulties, severe neurological/cardiac comorbidities, oxygen dependency and/or pulmonary arterial hypertension, for whom close supervision during CBPR would likely be most appropriate.

### Strengths and limitations

This study is the first to explore the perspectives of individuals with IPF regarding HBPR in India and identify considerations for HBPR in this context. The study involved three different groups of participants for a broader spectrum of views, knowledge, and experience.

Present study findings may only be applied to Delhi/National Capital Region and may not be representative of India due to its large population with various subcultures and practices across the country. Therefore, HBPR may look different and have different challenges in other regions of India. Furthermore, as we only recruited individuals with IPF within the present study, lived experiences and views towards HBPR may differ across other interstitial lung diseases.

Information relating to level of disease severity, oxygen use and digital skills, as well as HCWs years of experience in PR were unavailable. Results from the current study may differ between participants depending on their characteristics.

## Conclusion

Each participant group was positive about the development of HBPR to facilitate participation. The findings from this study suggest that HBPR in Delhi, India should be designed in a way that focuses mainly on visual representation with simple language, and should be sensitive to the additional impact of non-IPF health issues and challenges of reduced interactions with healthcare professionals.

## Supplemental Material

Supplemental Material - Understanding the lived experience of idiopathic pulmonary fibrosis and how this shapes views on home-based pulmonary rehabilitation in Delhi, India Supplemental Material for Understanding the lived experience of idiopathic pulmonary fibrosis and how this shapes views on home-based pulmonary rehabilitation in Delhi, India by Humaira Hanif, Obaidullah Ahmed, James Manifield, Rubia Ishrat, Ilaria Pina, Zahira Ahmed, Mohd Shibli, Dominic Malcolm, Deepak Talwar, Sally J Singh and Mark W Orme in Chronic Respiratory Disease.
