# Supplementary material for: Understanding the lived experience of idiopathic pulmonary fibrosis and how this shapes views on home-based pulmonary rehabilitation in Delhi, India
Source: Chron Respir Dis. 2024 May 24;21:14799731241258216. doi: 10.1177/14799731241258216 (PMC11127573; doi:10.1177/14799731241258216)
Supplement: Supplemental Material - Understanding the lived experience of idiopathic pulmonary fibrosis and how this shapes views on home-based pulmonary rehabilitation in Delhi, India [file sj-pdf-1-crd-10.1177_14799731241258216.pdf]

Supplementary material 1 - COREQ (COnsolidated criteria for REporting Qualitative research) Checklist

Topic	Item No.	Guide Questions/Description	Reported on Page No.
**Domain 1: Research team and reflexivity**			
Personal characteristics			
Interviewer/facilitator	1	Which author/s conducted the interview or focus group?	suppl
Credentials	2	What were the researcher's credentials? E.g. PhD, MD	suppl
Occupation	3	What was their occupation at the time of the study?	suppl
Gender	4	Was the researcher male or female?	suppl
Experience and training	5	What experience or training did the researcher have?	suppl
Relationship with participants			
Relationship established	6	Was a relationship established prior to study commencement?	5
Participant knowledge of the interviewer	7	What did the participants know about the researcher? e.g. personal goals, reasons for doing the research	5
Interviewer characteristics	8	What characteristics were reported about the inter viewer/facilitator? e.g. Bias, assumptions, reasons and interests in the research topic	5
**Domain 2: Study design**			
Theoretical framework			
Methodological orientation and Theory	9	What methodological orientation was stated to underpin the study? e.g. grounded theory, discourse analysis, ethnography, phenomenology, content analysis	6
Participant selection			
Sampling	10	How were participants selected? e.g. purposive, convenience, consecutive, snowball	4
Method of approach	11	How were participants approached? e.g. face-to-face, telephone, mail, email	suppl
Sample size	12	How many participants were in the study?	7
Non-participation	13	How many people refused to participate or dropped out? Reasons?	N/A
Setting			
Setting of data collection	14	Where was the data collected? e.g. home, clinic, workplace	6
Presence of non-participants	15	Was anyone else present besides the participants and researchers?	N/A
Description of sample	16	What are the important characteristics of the sample? e.g. demographic data, date	Table 1
Data collection			
Interview guide	17	Were questions, prompts, guides provided by the authors? Was it pilot tested?	5
Repeat interviews	18	Were repeat inter views carried out? If yes, how many?	N/A
Audio/visual recording	19	Did the research use audio or visual recording to collect the data?	6
Field notes	20	Were field notes made during and/or after the interview or focus group?	6
Duration	21	What was the duration of the inter views or focus group?	7
Data saturation	22	Was data saturation discussed?	No
Transcripts returned	23	Were transcripts returned to participants for comment and/or	No

Topic	Item No.	Guide Questions/Description	Reported on Page No.
		correction?	
**Domain 3: analysis and findings**			
Data analysis			
Number of data coders	24	How many data coders coded the data?	6
Description of the coding tree	25	Did authors provide a description of the coding tree?	No
Derivation of themes	26	Were themes identified in advance or derived from the data?	6
Software	27	What software, if applicable, was used to manage the data?	6
Participant checking	28	Did participants provide feedback on the findings?	No
Reporting			
Quotations presented	29	Were participant quotations presented to illustrate the themes/findings? Was each quotation identified? e.g. participant number	7-12
Data and findings consistent	30	Was there consistency between the data presented and the findings?	7-12
Clarity of major themes	31	Were major themes clearly presented in the findings?	7-12
Clarity of minor themes	32	Is there a description of diverse cases or discussion of minor themes?	7-12

Developed from: Tong A, Sainsbury P, Craig J. Consolidated criteria for reporting qualitative research (COREQ): a 32-item checklist for interviews and focus groups. *International Journal for Quality in Health Care*. 2007. Volume 19, Number 6: pp. 349 – 357

Supplementary material 2 - Methods

Participants

People living with IPF were recruited from an outpatient department (OPD) visit or via telephonic conversations, with patient data taken via medical records from the Metro Centre of Respiratory Diseases (MCRD), Noida, India. HCWs were recruited from the same hospital. CG were informed of the study during OPD visits with relatives with IPF.

Interview process

Topic guides were developed in collaboration with the researchers at University of Leicester, UK, and MCRD, India who have expertise in PR. The clinical experience of PR researchers at MCRD was also important to inform the development of topic guides and included conversations with individuals surrounding the challenges of living with IPF and their views of participating in centre-based PR programmes.

Interviews were conducted by two researchers (HH [PhD scholar, female], OA [MPT, male]), both physiotherapists at MCRD, with experience in delivering PR for people with IPF. They were trained in qualitative research data collection and analysis by authors with more experience (DM, MO) from University of Leicester and Loughborough University before conducting interviews. Support was provided throughout data collection and analysis.

Interviews were conducted in English, Hindi, or mixed as per participant preference, and were translated/transcribed in one common language (English) to make the process convenient for analysis. It was ensured that interpretation and meaning was maintained by the interviewer checking translated quotes against the original transcripts. Interviews were conducted either face-to-face or remotely, via telephone, due to COVID-19 restrictions.

Data Analysis

Codebook thematic analysis was conducted following the six stages of Braun and Clarke (15): (i) “Familiarization”: all transcripts were examined in detail by five researchers (HH, OA, RI, IP, MO); (ii) “Coding”: codes were generated by one (HH) researcher within Microsoft Excel, then arranged in hierarchal order to generate appropriate themes; (iii) “Generating initial themes”: potential themes were developed by one (HH) researcher by examining the codes; (iv) “Developing and reviewing themes”: initial themes were discussed by three researchers (HH, IP, MO) who ensured that these themes told the story of the data; (v) “Refining, defining, and naming themes”: a detailed analysis of each theme was performed by two researchers (HH and MO), who then mutually decided on informative names of themes; (vi) “writing up”: extracted data were interlaced together and contextualized the analysis according to existing literature and the local context by HH, with support from all authors.

Supplementary material 3 – Interview topic guides

IPF patient interview topic guide	
**Topic**	**Questions/prompts**
Experiences and impact of living with IPF	*How did you expect IPF was going to affect you?*<br>*Did you know about your disease IPF such as how it affects you, what precaution or care you have to take?*<br>*Did you know the types of ILD, and what type you have?*<br>*Did your doctor tell you about your condition, and did they explain to you about IPF progression and impact on your health and functional capacity?*<br>*What would you list as the most significant thing about this condition? In what ways? (Prompts: personally/professionally; impact on your life/ activities in general etc.)*<br>*Has IPF had a significant impact on your relations with other people? Who has helped you or not helped you? How?*<br>*How has your work (employment) changed?*<br>*How has IPF affected your domestic duties? (Prompts: Is there anything you do less or more of?)*<br>*How has IPF affected your leisure activities? (Prompts: Have you taken up new ones?)*<br>*What would you advise others in your situation to do? What would you advise others to not do or what situations/tasks to avoid?*
Physical activity/exercise	*When are you most active in the day?*<br>*What physical activities would you be happy doing more of?*<br>*What physical activities would you not be happy doing more of?*<br>*Would you like to include any other activities into your routine? What activities? Is there anything you would need to change to be able to include these activities?*
Experiences with pulmonary rehabilitation (PR) and home-based PR (HBPR) suggestions	*Have you heard about or know about pulmonary rehabilitation?*<br>*Do you know about the mode of deliveries of pulmonary rehabilitation like HBPR or tele-rehabilitation?*<br>*What would you expect to be included in a HBPR programme? What would you like to be included (Prompts: physical activity and exercise, diet, mental wellbeing, hobbies, relationships, medication)? Is there anything else you would like to be included?*

	What would you expect to not be included?<br>*How would you hope the HBPR might help you?*<br>*What would you hope to achieve from HBPR?*<br>*What challenges or concerns might you have about a HBPR programme?*<br>*What support, if any, would you expect to have during the HBPR programme?*<br>*(Prompts: from family, friends, doctors, nurses)*

Family caregivers (CGs) interview topic guide	
**Topic**	**Questions/prompts**
Experiences and impact caring for someone with IPF	*How did you react when you were told your family member has IPF? How did you feel?*<br>*How do you help your family member with their condition?*<br>*What has been the most significant change for your family member?*<br>*Is there anything you wish you had been told about IPF in order to help care for your family member? If so, what? How would this have helped you? (Prompts: Lungs, Activity, Diet, symptoms, medication, causes).*<br>*How would you like this information to look? How should it be made available to people? (Prompts: paper-based or online?)*
Attitudes towards physical activity/exercise	*What do you think about your relation's day to day physical activity?*<br>*According to your perspective, what will be the most effective ways in improving physical activity of your caretaker's/ relation's?*
PR recommendations	*Do you think relations/caregivers should be involved during PR? How? Why?*<br>*What will be the most possible and beneficial method and time in delivering PR for your relative?*<br>*What do you think are the barriers and obstacles you may face when your caretaker's/ relations are following PR? (Prompt: COVID – safety considerations).*<br>*What do you think should be included in a HBPR programme? Are there any activities your family member might enjoy? Any activities which might help them?*<br>*Do you have any suggestions in regards to the HBPR programme?*

Healthcare workers (HCW) interview topic guide	
**Topic**	**Questions/prompts**
PR knowledge and experience	*Do you know about PR?*<br><br>*What experience do you have of running a PR programme?*<br><br>*How important do you think it is to provide PR?*<br><br>*How does PR compare to other forms of treatment such as stopping smoking, reduce biomass exposure, medication, etc.?*
HBPR views	*What do you think about a HBPR programme? How do you feel about patients doing PR at home?*<br><br>*How do you think the effects may be different to PR at a centre?*<br><br>*How do you feel about referring patients to HBPR? (Prompts: do you have any concerns?)*<br><br>*How would you feel answering questions from the patients regarding HBPR?*<br><br>*What do you think the challenges of HBPR will be? (Prompts: facilities, patients, working with colleagues, particular aspects of the PR programme, communication gap, counselling of patients and their relatives)*<br><br>*How do you think patients will perceive HBPR? (Prompts: compared to other forms of treatment such as stopping smoking, reduce biomass exposure, medication, etc., compared to centre-based PR)*
HBPR recommendations	*What physical activities/exercise are good for these patients? Why? How might we incorporate these activities as part of HBPR?*<br><br>*What do you think should be included in HBPR? (Prompts: Education, information, types of exercises [e.g., walking]) Why? Why not other activities?*

Supplementary material 4 – Further quotes and interpretations

Health impact

Impact of IPF symptoms on daily life

Negative thoughts and mood swings were reported among individuals with IPF due to coping with a progressive disease, "*I have become anxious and irritable after illness, sometime I have bad situation because of my irritability but my children know this and they understand my situation. I became stubborn and reluctant which I should not"" (P1020).*

Home Based PR/ Mode of PR

HBPR advantages:

Furthermore, it was suggested that HBPR could overcome the barrier of individuals with IPF relying on CGs to take them to rehabilitation sessions and fitting this around their busy schedules, "*Sometimes I have to reschedule my meetings, or I have to ask my elder brother to come in […] I hope the manual will itself help to overcome that" (C1002).*

Challenges with HBPR:

HCWs concern was more towards compliance, techniques, and exercise progression, *"No motivation, no physiotherapist who can guide them throughout the rehabilitation process. We can also not assess our patients progress on daily basis. Without us most of the time it is difficult for the patient to do exercise with coordinated and correct movement (H1002).*
